# A Novel Alginate Lyase: Identification, Characterization, and Potential Application in Alginate Trisaccharide Preparation

**DOI:** 10.3390/md20030159

**Published:** 2022-02-23

**Authors:** Zhao Xue, Xiao-Meng Sun, Cui Chen, Xi-Ying Zhang, Xiu-Lan Chen, Yu-Zhong Zhang, Shou-Jin Fan, Fei Xu

**Affiliations:** 1 Life Science College, Shandong Normal University, Jinan 250014, China; zxue@qnlm.ac (Z.X.); 11210611023@stu.ouc.edu.cn (X.-M.S.); CC20220122@163.com (C.C.); zhangyz@sdu.edu.cn (Y.-Z.Z.); 2 College of Marine Life Sciences, and Frontiers Science Center for Deep Ocean Multispheres and Earth System, Ocean University of China, Qingdao 266003, China; 3 State Key Laboratory of Microbial Technology, Marine Biotechnology Research Center, Shandong University, Qingdao 266237, China; zhangxiying@sdu.edu.cn (X.-Y.Z.); cxl0423@sdu.edu.cn (X.-L.C.); 4 Laboratory for Marine Biology and Biotechnology, Pilot National Laboratory for Marine Science and Technology (Qingdao), Qingdao 266237, China

**Keywords:** alginate lyase, alginate oligosaccharides, trisaccharide, PL6 lyase, *Pseudoalteromonas*

## Abstract

Alginate oligosaccharides (AOS) have many biological activities and significant applications in prebiotics, nutritional supplements, and plant growth development. Alginate lyases have unique advantages in the preparation of AOS. However, only a limited number of alginate lyases have been so far reported to have potentials in the preparation of AOS with specific degrees of polymerization. Here, an alginate-degrading strain *Pseudoalteromonas* *arctica* M9 was isolated from *Sargassum*, and five alginate lyases were predicted in its genome. These putative alginate lyases were expressed and their degradation products towards sodium alginate were analyzed. Among them, AlyM2 mainly generated trisaccharides, which accounted for 79.9% in the products. AlyM2 is a PL6 lyase with low sequence identity (≤28.3%) to the characterized alginate lyases and may adopt a distinct catalytic mechanism from the other PL6 alginate lyases based on sequence alignment. AlyM2 is a bifunctional endotype lyase, exhibiting the highest activity at 30 °C, pH 8.0, and 0.5 M NaCl. AlyM2 predominantly produces trisaccharides from homopolymeric M block (PM), homopolymeric G block (PG), or sodium alginate, with a trisaccharide production of 588.4 mg/g from sodium alginate, indicating its promising potential in preparing trisaccharides from these polysaccharides.

## 1. Introduction

Alginate is an acidic linear polysaccharide present in great abundance in the cell wall of brown algae [1], accounting for approximately 30–60% dry weight of brown algae [2]. In addition, alginate also exists in some red algae [3] and can be produced by several bacteria, such as genera *Azotobacter* and *Pseudomonas* [4]. Alginate is composed of β-D-mannuronate (M) and its C5 epimer, α-L-guluronate (G), which are linked by 1,4-O-glycoside bonds and arranged in block structures, such as homopolymeric G block (PG), M block (PM), and heteropolymeric MG/GM block [5]. Alginate possesses unique physical properties, such as viscosity, sol/gel transition, and water uptake properties, which contribute to its various applications as stabilizers, emulsifiers, thickeners, and gel-forming agents in the food, cosmetic, and pharmaceutical industries [1].

Alginate oligosaccharides (AOS), the degradation products of alginate, have received increasing attention due to their low molecular weights (MWs) and promising biological activities, including antitumor (MW_AOS_: 414–2161 Da) [6], antioxidant (MW_AOS_: ~1500 Da) [7], antibacterial [8], hypotensive (MW_AOS_: ~500 Da) [9], hypoglycemic (MW_AOS_: ~5000 Da) [10], neuroprotection [11], immunomodulatory (MW_AOS_: 414–1602 kDa) [12], promoting cellular proliferation and regulating plant growth activities (MW_AOS_: 414–865 kDa) [13]. Hence, AOS have significant applications in prebiotics, nutritional supplements, plant growth development and others products [14]. In addition, oligosaccharides of various degrees of polymerization (DPs) have been shown to have various effects. For instance, the unsaturated DP3–9 oligomers could induce tumor necrosis factor (TNF)-α in a structure-dependent manner, with octaguluronate and heptamannuronate showing the most potent activity [15]. AOS mainly composing of trisaccharides can regulate plant growth [16], attenuate spontaneous hypertension [17], and have the prebiotic potential to stimulate the growth of bifidobacteria or lactobacilli [18]. A variety of physical, chemical, and enzymatic methods have been applied to produce AOS. The physical methods include gamma ray [19], ultraviolet irradiation [20], and ultrasound [21]. The chemical methods include hydrochloric acid [22] and oxidative degradation [23]. Enzymatic methods, mainly through alginate lyases, are widely used in alginate degradation due to its advantages of high efficiency and specificity, safety, and environmental friendliness [24,25].

Alginate lyases degrade alginate by a β-elimination mechanism, generating a product containing 4-deoxy-L-erythro-hex-4-enopyranosyluronic acid as the non-reducing terminal moiety [26]. Based on the protein sequences, alginate lyases are classified into the polysaccharide lyase (PL) 5, −6, −7, −8, −14, −15, −17, −18, −31, −32, −34, −36, −39 and −41 families in the Carbohydrate-Active enZYmes (CAZy) database [2,27]. The three-dimensional structures of some representative alginate lyases from these families (except PL32 and PL34) have been solved, which can be grouped into four categories: single domain (α/α)_n_ toroid (PL5), single domain β-jelly roll (PL7, PL14, PL18 and PL36), right-handed β-helix (PL6 and PL31), and multidomain (α/α)_n_ toroid + antiparallel β-sandwich (PL8, PL15 and PL17) structures [2,28]. DP0100 from PL39 contains a more complex structure composed of three domains, an N-terminal incomplete (α/α)_6_ toroid, two β-sheets with a distorted α-helix in the central region, and a typical β-sandwich in the C-terminus [29]. Alginate lyases are classified into three groups according to their substrate specificity: the first type is specific toward G block (EC 4.2.2.11), the second type is specific toward M block (EC 4.2.2.3), and the third type is bifunctional for G and M blocks (EC 4.2.2.-) [1]. According to the action mode, alginate lyases are grouped into exotype lyases producing monomers and endotype lyases that generate AOS mainly ranging from DP2-DP5 [2]. However, only a limited number of alginate lyases have been so far reported to have potentials in the preparation of AOS with specific DPs [30,31,32]. Thus, it is necessary to find out more such alginate lyases for the production of specific DPs.

In this study, an alginolytic strain, *Pseudoalteromonas arctica* M9, was isolated from *Sargassum*, and five genes encoding alginate lyases were predicted based on analyzing its genome. These putative alginate lyases were heterologously expressed, and their degradation products released from sodium alginate were determined. Among them, AlyM2 was found to specifically produce trisaccharides. Sequence analysis and biochemical characterization suggested that AlyM2 is a novel alginate lyase of the PL6 family, which has good potential in preparing various alginate trisaccharides.

## 2. Results and Discussion

### 2.1. Screening and Identification of Strain M9

*Sargassum* species are the largest canopy-forming brown algae, which are widely distributed in tropical and subtropical environments and have high amount of biomass [33]. Due to the high content of alginate (>50% of their dry weight) [34], *Sargassum* species are good materials to isolate alginate lyase-excreting bacteria. Thus, we collected *Sargassum* samples from coastal seawaters to isolate alginate lyase-excreting bacteria.

**Figure 1:**
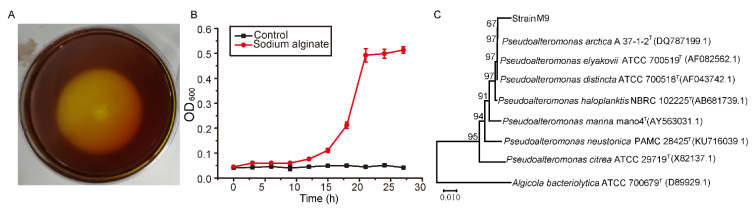
Screening of strain M9. (**A**), the plate assay of strain M9. The plate containing sodium alginate as the sole carbon source was stained by Lugo’s iodine solution to detect the appearance of a clear halo of depolymerization around the strain colony. (**B**), the growth curve of strain M9 cultured in the liquid minimal medium containing 0.5% sodium alginate as the sole carbon source. The growth curve of strain M9 cultured in the liquid minimal medium without sodium alginate was taken as the control. (**C**), the neighbor-joining phylogenetic tree of strain M9 based on the 16S rRNA gene sequences. The bootstrap values of each branch were tested by 1000 repetitions.

After seven-day cultivation of the bacteria from the *Sargassum* samples on the plates, many colonies appeared on the plates. Alginate lyase-excreting bacteria were further screened from the *Sargassum*-associated strains by plate assay. After four-day cultivation, an isolate, which was named strain M9, was found to produce a clear halo with the diameter ratio of the halo to the colony of 8 (Figure 1A), suggesting that strain M9 may secrete alginate lyases to degrade the alginate in the medium. We then inoculated strain M9 in the minimal medium with alginate as the sole carbon source to further confirm this. After 24 h cultivation, the OD_600_ of the culture increased 0.45 (Figure 1B), and the extracellular activity of the culture toward sodium alginate was 0.55 ± 0.01 U/mL when detected by the 3,5-dinitrosalicylic acid (DNS) method. In contrast, cells in the minimal medium without alginate showed almost no increase in 24 h (Figure 1B). These results indicated that strain M9 is an alginate lyase-excreting bacterium, which utilizes alginate for growth via secreting alginate lyases to degrade alginate.

To identify strain M9, the 16S rRNA gene (1463 bp) of strain M9 was amplified and sequenced, and a phylogenetic analysis was performed. The result showed that strain M9 was clustered with many *Pseudoalteromonas* species, most close to *Pseudoalteromonas arctica* A 37-1-2^T^ (Figure 1C). Therefore, strain M9 belongs to the genus *Pseudoalteromonas,* and was identified as an alginate-degrading strain member of *Pseudoalteromonas arctica*, named as *Pseudoalteromonas arctica* strain M9.

*Pseudoalteromonas* contains 48 species of heterotrophic bacteria that are widely distributed in marine environments and are an important group in alginate degradation [35]. However, to date, no alginate-degrading *Pseudoalteromonas* strains have been isolated from *Sargassum.* The isolation and identification of strain M9 from *Sargassum* is helpful to excavate alginate lyases and alginate metabolism related genes.

### 2.2. Bioinformatic Analysis of the Alginate Lyases of Strain M9

**Figure 2:**
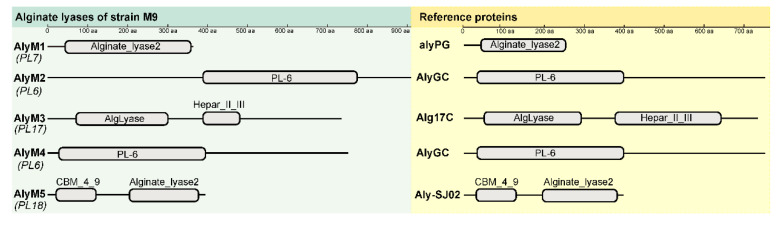
Domain architectures of the alginate lyases of strain M9. The domain structures were drawn based on the prediction on the Conserved Domain Database (https://www.ncbi.nlm.nih.gov/Structure/cdd/wrpsb.cgi; accessed on 17 December 2021). Only conserved domains predicted in the sequences are shown. The lines in the domain architecture diagram represent the sequence regions not annotated as any conserved domain. The reference proteins were selected from the corresponding PL families of each alginate lyase of strain M9, including alyPG (BAA83339.1) of PL7, AlyGC (WP_007984897.1) of PL6, Alg17C (ABD82539.1) of PL17 and Aly-SJ02 (ACB87607.2) of PL18.

To identify the alginate lyases in strain M9, its genome was sequenced and analyzed. In the genome, five genes were predicted to encode alginate lyases. As shown in Table 1, these putative alginate lyases, named AlyM1, AlyM2, AlyM3, AlyM4, and AlyM5, belong to four different PL families. AlyM1 belongs to the PL7 family and has only one domain (Figure 2). AlyM3 and AlyM5, belonging to the PL17 and PL18 families, respectively, both have two domains, which are conventional in their corresponding families (Figure 2). AlyM2 and AlyM4, both belonging to the PL6 family, have different modularity. The catalytic PL6 domain of AlyM2 is at the C-terminus and that of AlyM4 at the N-terminus (Figure 2). The five alginate lyases all have predicted signal peptides, suggesting that they are likely extracellularly secreted enzymes.

### 2.3. Expression and Degradation Product Analysis of the Alginate Lyases of Strain M9

**Figure 3:**
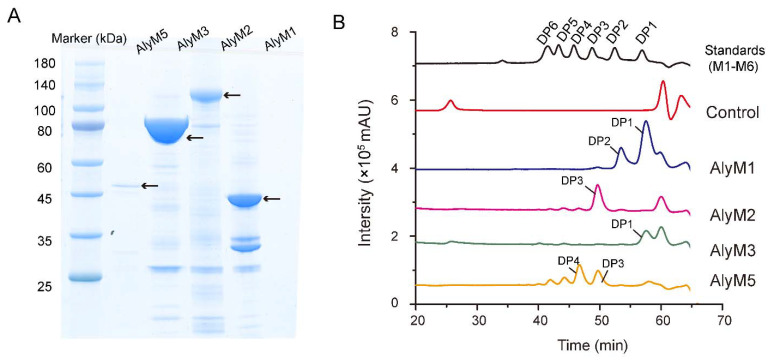
Degradation products analysis of the alginate lyases of strain M9. (**A**), SDS-PAGE analysis of the purified alginate lyases AlyM1, AlyM2, AlyM3, and AlyM5. The recombinant protein bands of the alginate lyases are indicated by arrows. (**B**), gel filtration chromatography analysis of the degradation products of the alginate lyases. Gel filtration chromatography analysis was performed using a Superdex peptide 10/300 GL column monitored at a wavelength of 210 nm. The control was treated with pre-heated inactivated lyases. Saturated mannuronate oligosaccharides from DP1 to DP6 were taken as the standards. DP, degree of polymerization.

To evaluate the application potentials of the alginate lyases of strain M9 in AOS preparation, we expressed all the five putative alginate lyases in *Escherichia coli* with His-tag and purified them by NTA-Ni Sepharose affinity chromatography. Finally, AlyM1, AlyM2, AlyM3, and AlyM5 were successfully purified (Figure 3A), but AlyM4 failed. Although many attempts were made to improve the expression amount via optimizing the conditions for its expression, the expression amount of AlyM4 was still too low to be purified. To obtain the end products of the four purified enzymes towards sodium alginate, each enzyme was incubated with sodium alginate at 30 °C for 12 h. The degradation products were then determined by gel filtration assay (Figure 3B). The result showed that AlyM3 is an exotype lyase producing monomer as the sole product, and AlyM1, AlyM2, and AlyM5 are endotype lyases producing different AOS products. AlyM1 produced mainly monosaccharide and disaccharide, with a small amount of trisaccharide. The degradation products of AlyM5 were a mixture of DP3-DP6 saccharides. In contrast, in addition to a slight amount of DP2 and DP4-DP6, the degradation products of AlyM2 are mainly trisaccharides. Trisaccharides accounted for 79.9% of the product amount based on the peak area of the products in the gel filtration chromatogram (total peak area of all products, ~31,380,656; the peak area of trisaccharides, 25,073,144).

In previous studies, the products of endotype alginate lyases mainly range from DP2 to DP5, and only a limited number of alginate lyases have been shown to have potentials in the preparation of AOS with specific DPs. While ALFA3, Aly1281, AlySY08, and Val-1 were shown to be suitable for disaccharide production [30,31,36,37], Alg7A, AlyF, and AkAly28 were reported to mainly produce trisaccharides [32,38,39]. The high proportion of trisaccharides in the products of AlyM2 implies that AlyM2 may have potential in trisaccharide production. Thus, we further characterized AlyM2 and evaluated its potential in preparing alginate trisaccharides.

### 2.4. Sequence Analysis and Structural Prediction of the Alginate Lyase AlyM2

**Figure 4:**
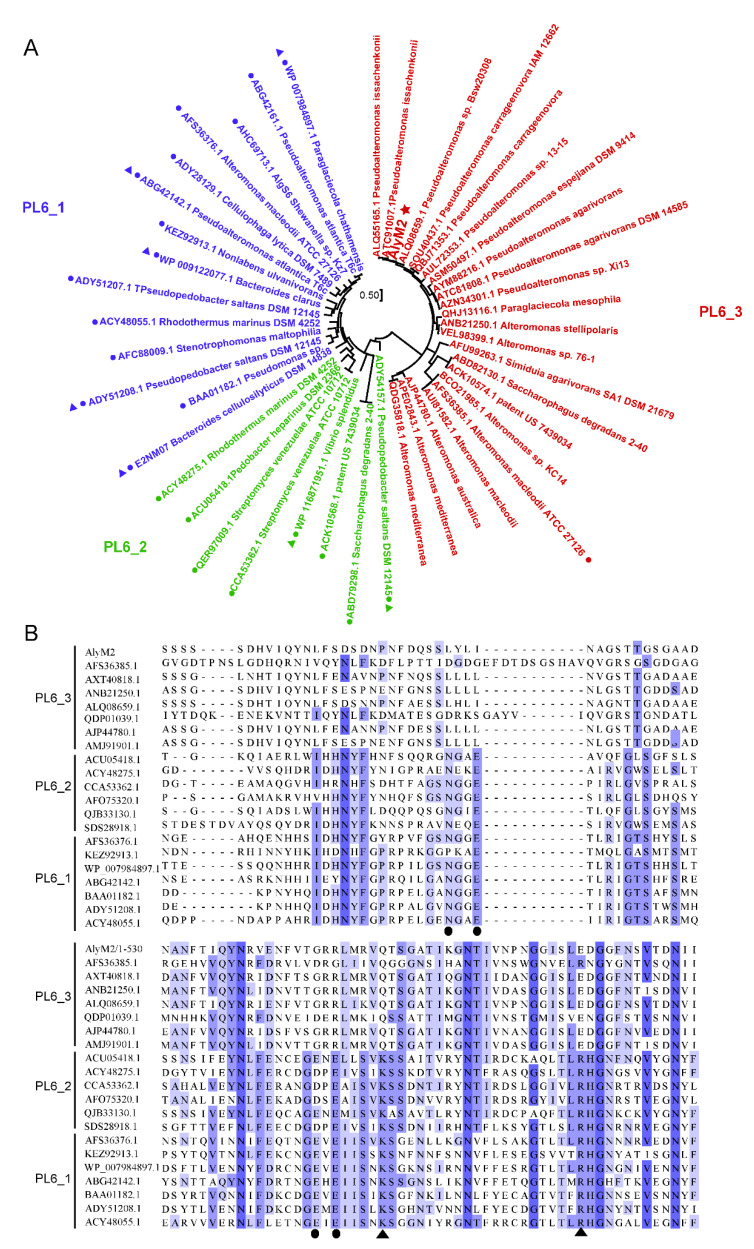
Sequence analysis of AlyM2. (**A**), phylogenetic analysis of the alginate lyases from the PL6 family by using the maximum likelihood method. The alginate lyases of subfamily PL6_1 are in blue, those of PL6_2 in green and those of PL6_3 in red. AlyM2 is marked with a star. The characterized alginate lyases are marked with spots. The structure-solved alginate lyases are marked with triangles. (**B**), multiple sequence alignment of AlyM2 and other alginate lyases of the PL6 family. The amino acids involved in catalysis and those involved in coordination with water or metal ions are marked with triangles and spots, respectively. Identical and similar amino acid residues among the enzymes are shaded in blue.

AlyM2 is a PL6 alginate lyase containing 910 amino acid residues (Table 1). Alginate lyases in the PL6 family are divided into three subfamilies, that is, PL6_1, PL6_2, and PL6_3. Based on phylogenetic analysis, AlyM2 is a PL6_3 lyase, clustered with the only characterized PL6_3 alginate lyase MASE_04180 from *Alteromonas macleodii* ATCC 27126 [40] and many predicted PL6_3 alginate lyases in the CAZy database (Figure 4A). AlyM2 exhibited a quite low sequence identity (28.3%) with MASE_04180 (Table 1).

The catalytic mechanisms of some PL6 alginate lyases have been revealed [40]. In these PL6 enzymes, a conserved lysine and a conserved arginine usually act as the two catalytic residues, and several conserved residues interact with the water molecule or calcium ion to neutralize the negative charge of the carboxyl group [40]. However, multiple sequence alignment of AlyM2 with alginate lyases from three PL6 subfamilies showed that the amino acid of PL6_3 alginate lyases corresponding to the reported catalytic lysine of other enzymes is glutamine, and the amino acid of most PL6_3 alginate lyases corresponding to the catalytic arginine is aspartic acid (Figure 4B). Moreover, the amino acids coordinated with water or Ca^2+^ in PL6_3 alginate lyases are likely different from those in other PL6 enzymes (Figure 4B). These data suggest that AlyM2 and other PL6_3 alginate lyases may have a potentially distinct catalytic mechanism from those of PL6_1 and PL6_2, which awaits further confirmation.

**Figure 5:**
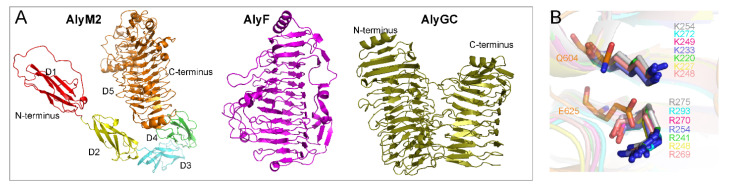
Structure comparison of AlyM2 with other PL6 alginate lyases. (**A**), comparison of the overall structure of AlyM2 with other PL6 structures. AlyM2 structure was predicted by Alphafold2. Structures of AlyF (Genbank accession number: WP_116871951.1; PDB code: 6ITG) and AlyGC (Genbank accession number: WP_007984897.1; PDB code: 5GKQ) are representative PL6 structures. (**B**), the arrangement of the key residues in the active centers of the PL6 alginate lyases. The amino acid residues are shown in sticks. AlyM2 is in orange, Patl_3640 (PDB code: 7O77) in silver, Pedsa_3628 (PDB code: 7O79) in blue, Pedsa_0632 (PDB code: 7O7A) in salmon, AlyGC (PDB code: 5GKD) in green, AlyF (PDB code: 5Z9T) in cyan, BcelPL6 (PDB code: 6QPS) in magenta, and BcAlyPL6 (PDB code: 7DML) in yellow.

Among PL6 alginate lyases, the structures of seven enzymes have been reported, including five from PL6_1 and two from PL6_2 (Figure 4A). These structures can be divided into two kinds. Some, represented by AlyF (PDB code: 6ITG), contain only a catalytic domain exhibiting the β–helix fold [38]. The others, represented by AlyGC (PDB code: 5GKQ), contain two domains both exhibiting the β–helix fold, and their N-terminal domain is the catalytic domain [41] (Figure 5A). The overall structure of AlyM2 was predicted by Alphafold2 [42], which suggests that AlyM2 consists of five domains, including domain 1 (D1), domain 2 (D2), domain 3 (D3), domain 4 (D4), and domain 5 (D5) from the N-terminus to the C-terminus (Figure 5A). The C-terminal D5 exhibits the typical β–helix fold of the catalytic domains of the reported PL6 alginate lyases [38,41], suggesting that D5 is likely the catalytic domain of AlyM2. Domains D1, D2, D3, and D4 all consist of five or six β strands. Thus, the overall structure of AlyM2 may be quite different from the reported PL6 alginate lyase structures, which awaits further investigation. Furthermore, an alignment of the predicted AlyM2 structure with seven reported PL6 structures showed that a glutamine and an aspartic acid of AlyM2 spatially correspond to the known catalytic lysine and arginine of these alginate lyases, respectively (Figure 5B). This is consistent with the above sequence alignment result (Figure 4B), further suggesting that AlyM2 and other PL6_3 alginate lyases may have a different catalytic mechanism from those of PL6_1 and PL6_2.

### 2.5. Biochemical Characterization of AlyM2

**Figure 6:**
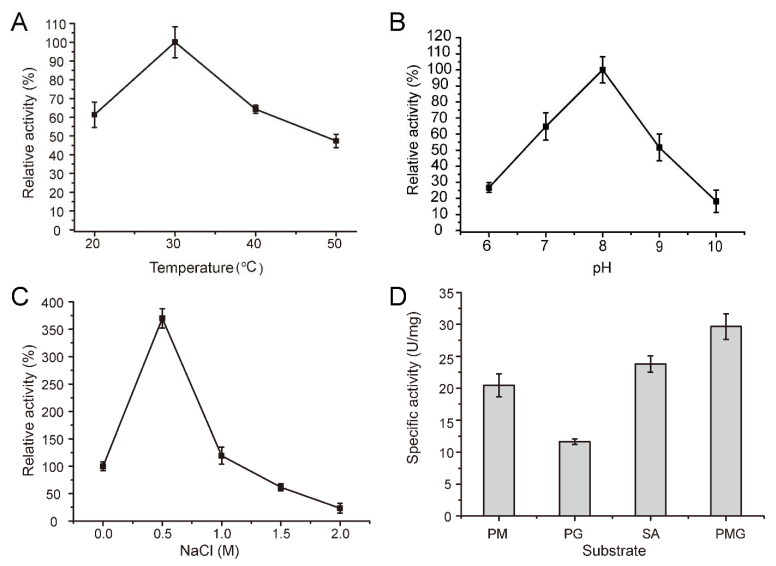
Biochemical characterization of AlyM2. (**A**), effect of temperature on AlyM2 activity. The highest activity at 30 °C was taken as 100%. (**B**), effect of pH on AlyM2 activity. The highest activity at pH 8.0 was taken as 100%. (**C**), effect of NaCl concentration on AlyM2 activity. The activity at 0 M NaCl is taken as 100%. (**D**), the substrate specificity of AlyM2 towards PM, PG, PMG, and sodium alginate (SA).

The recombinant AlyM2 was further biochemically characterized. As shown in Figure 6A, AlyM2 exhibited the maximum activity at 30 °C. Its activity was more than 60% of the maximum activity at 20 °C, and less than 50% at 50 °C. The activity of AlyM2 was the highest at pH 8.0 and maintained higher than 50% at a range of pH 7.0–9.0. At pH 10.0, the enzyme activity dropped to 18.2% (Figure 6B). Low NaCl concentrations less than 1 M significantly promoted AlyM2 activity. In particular, 0.5 M NaCl increased the activity of AlyM2 by nearly 4 times compared with that without NaCl (Figure 6C), indicating that AlyM2 has a salt-activated characteristic. These characteristics reflect the adaptation of AlyM2 to the seawater environment.

To investigate the substrate specificity of AlyM2, we measured the activity of AlyM2 towards four alginate substrates, PM, PG, PMG, and sodium alginate. AlyM2 had activity towards all these substrates (11.7–29.7 U/mg). Among them, its activity towards PMG was the highest (29.7 U/mg), and that towards PG was the lowest (11.7 U/mg) (Figure 6D). This result suggested that AlyM2 is capable of cleaving the M-M bond, the G-G bond, and the M-G bond in the substrates. To date, only one PL6_3 alginate lyase, MASE_04180, has been characterized, which can cleave only the M-G bond [40]. Thus, AlyM2 is distinct from MASE_04180 in substrate specificity.

### 2.6. Evaluation of the Potential of AlyM2 in Alginate Trisaccharides Preparation

**Figure 7:**
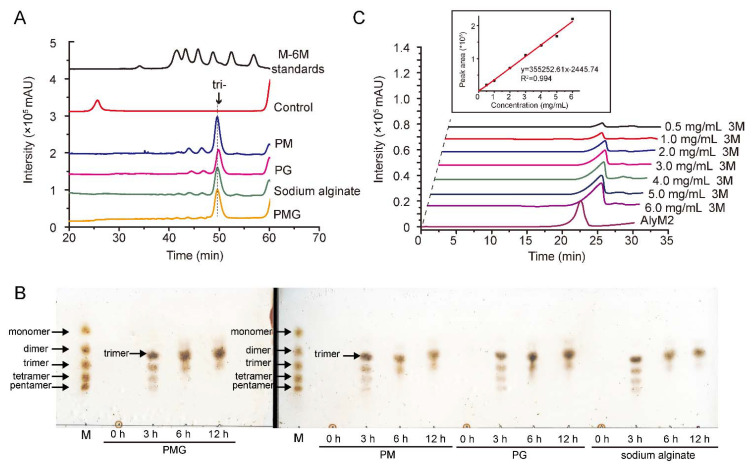
The production of trisaccharide from alginate substrates by AlyM2. (**A**), the end degradation products of AlyM2 towards PM, PG, PMG, and sodium alginate. A 200 μL mixture containing 80 μg/mL AlyM2 and 2 mg/mL substrate was incubated at 30 °C for 12 h in the buffer containing 50 mM Tris-HCl (pH 8.0) and 0.5 M NaCl. The degradation products were analyzed by gel filtration chromatography and monitored at 210 nm using a UV detector. Saturated mannuronate oligosaccharides from DP1 to DP6 were taken as the standards. (**B**), the effect of degradation time on the products. The reactions were carried out under the same conditions as described in Figure 7A for 0, 3, 6, or 12 h. The degradation products were analyzed by TLC. Lane M, saturated oligosaccharides standards including monomer, dimer, trimer, tetramer, and pentamer. (**C**), determination of the production of trisaccharides from sodium alginate degradation by AlyM2. The degradation reaction was performed in a 500 mL reaction mixture containing 1 g sodium alginate and 40 mg AlyM2 in the buffer containing 50 mM Tris-HCl (pH 8.0) and 0.5 M NaCl at 30 °C for 6 h. The degradation products were separated by gel filtration and monitored using a refractive index detector. A standard curve of trisaccharide concentration versus peak area was drawn by using commercial saturated mannuronate trisaccharide at the concentrations of 0–6 mg/mL as standards. The trisaccharide amount released from sodium alginate by AlyM2 was determined based on the standard curve.

We further analyzed the end degradation products of AlyM2 towards PM, PG, and PMG. Similar to the result of AlyM2 on sodium alginate (79.9% trisaccharides in the products), trisaccharides were also the main component of the degradation products (Figure 7A), accounting for 78.2% in those of PM, 79.6% in those of PG, and 76.75% in those of PMG. This result suggested that AlyM2 can be used to produce different alginate trisaccharides, including trimannuronate from PM, triguluronate from PG, and a mixture of trisaccharides from PMG and sodium alginate.

To investigate the effect of degradation time on the products, the four alginate substrates were degraded with AlyM2 for different times from 0 to 12 h, and the degradation products were detected by thin layer chromatography (TLC). As shown in Figure 7B, after 3 h degradation, the products from all the alginate substrates were a mixture of oligosaccharides with DP3-DP6; after 6 h degradation or more time, trimer was the main product, with only a small amount of tetramer and a negligible amount of dimer. Therefore, the degradation time can be shortened to 6 h.

By using commercial saturated mannuronate trisaccharides as standards, we further calculated the production of trisaccharides when sodium alginate was degraded by AlyM2, which was determined to be 588.4 mg/g sodium alginate (Figure 7C).

The biological activity of AOS is closely related to its DP [43]. It has been reported that AOS with an average DP of 3, including ΔGG and a mixture of ΔMM and ΔMM’ (the β anomer of M), have conspicuous root growth-promoting activities in barley seedlings [16], and treatment with sodium AOS of DP3 (3 α-L-guluronate and/or β-D-mannuronate) can attenuate spontaneous hypertension [17]. Till now, according to the summary from Cheng et al., 3 alginate lyases have been shown to mainly produce trisaccharides [2], that is, AlyF from *Vibrio splendidus* OU02 [38], Alg7A from *Vibrio* sp. W13 [32] and AkAly28 from *Aplysia kurodai* [39] (Table 2). AlyF is a PG specific lyase, and AkAly28 is a PM lyase, both of which are distinct from AlyM2 in substrate specificity. Alg7A is similar to AlyM2 in substrate specificity, having activity towards PM, PG, PMG, and sodium alginate [32]. While trimers are the predominant products of Alg7A towards all the four substrates, dimers and tetramers are also the main products [32] (Table 2). However, in the degradation products of AlyM2 towards the four substrates, trimers are the main products, with only a small amount of DP4 and a negligible amount of DP2 (Figure 7B). Therefore, AlyM2 is promising for the preparation of different alginate trisaccharides with high purity. Since AOS of DP3 have been reported to have many bioactivities [16,17,18], AlyM2 may have application potential in the production of trisaccharides with healthcare potential and other industrial applications, such as treatment of hypertension, acting as plant growth regulators and healthcare supplements, which awaits further investigation.

## 3. Materials and Methods

### 3.1. Materials and Strains

Sodium alginate derived from brown seaweed was purchased from Sangon (Shanghai, China). PM, PG (6–8 kDa), and alginate oligosaccharides (purity ≥ 97%) were purchased from Zzstandard (Qingdao, China). PMG was prepared as previously described [45]. Other chemicals and reagents used in this study are of analytical grade. *E. coli* strains were from TransGen (Beijing, China) and cultured in Lysogeny broth (LB) medium at 37 °C.

### 3.2. Screening of Strain M9

*Sargassum* samples were collected from coastal seawaters in Shandong Province, China (37°9′41.7″ N, 122°35′11.8″ E) in March 2019 and suspended in sterile artificial seawater (ASW) prepared with 3% (*w/v*) sea salts (Sigma, Saint Louis, MO, USA). The obtained suspension was diluted to10^−2^–10^−6^ dilution, and then 200 μL of each diluted solution was spread on the plates containing the Zobell agar medium composed of 0.5% (*w/v*) tryptone (Oxoid, Basingstoke, UK), 0.1% (*w/v*) yeast extract (Oxoid, Basingstoke, UK), 1.5% (*w/v*) agar (Sigma, Saint Louis, MO, USA), and ASW. The plates were incubated at 15 °C for 7 days to obtain morphologically different colonies. The purified isolates were streaked on the plates with a minimal medium (0.05% NH_4_Cl, 3% NaCl, 0.3% MgCl_2_·6H_2_O, 0.2% K_2_SO_4_, 0.02% K_2_HPO_4_, 0.001% CaCl_2_, 0.0006% FeCl_3_·6H_2_O, 0.0005% Na_2_MoO_4_·7H_2_O, 0.0004% CuCl_2_·2H_2_O, 0.6% Tris (pH 7.5–8.0), and 1.5% agar) containing 0.5% sodium alginate as the sole carbon source. After incubation at 25 °C for 7 days, the plates were stained by Lugo’s iodine solution to detect the appearance of a clear halo of depolymerization around a strain colony as a preliminary indicator of alginate degradation. Then, strains with a clear halo were purified, inoculated into the liquid minimal medium containing 0.5% sodium alginate, and cultured at 20 °C with stir (180 rpm) for 24 h. Strain M9 that formed a clear depolymerization halo on the plate and grew noticeably in the liquid medium was chosen as an alginate-degrading strain for further study.

### 3.3. Determination of Extracellular Alginate Lyase Activity

Extracellular alginolytic activity of strain M9 was quantitatively determined by measuring the amount of reducing sugars released from sodium alginate by using the DNS method [46,47]. The fermentation broth of strain M9 cultured at 25 °C with stir (180 rpm) for 24 h was centrifuged at 15,871× *g* for 5 min to obtain the supernatant as crude enzyme. Then, 50 μL crude enzyme was mixed with 50 μL substrate solution composed of 1% (*w/v*) sodium alginate dissolved in 50 mM Tris–HCl (pH 8.0), and the mixture was incubated at 30 °C for 60 min. Afterwards, the reaction mixture was terminated by the addition of 100 μL DNS and then boiled at 100 °C for 10 min. A blank control was set by mixing DNS solution and the substrate solution followed by an addition of crude enzyme to inactivate the alginate lyase activity before incubation at 30 °C. The absorbance values of the mixtures were determined at 540 nm. The amount of reducing sugars released into the mixture was determined with glucose as the standard. One unit of enzyme activity is defined as the amount of enzyme required to release 1 µg reducing sugars from sodium alginate per min.

### 3.4. Sequencing of the 16S rRNA Gene and the Genomic DNA of Strain M9

Genomic DNA of strain M9 was extracted using a BioTekeDNA extraction kit (Beijing, China). The 16S rRNA gene was amplified from the genome DNA via PCR using the primers 1492R and 27F [26] (Table 3). The PCR product was ligated to the pMD19-T vector (TaKaRa, kusatsu, Japen) and sequenced on an Applied Biosystems DNA sequencer (model 3730XL). The sequence of the 16S rRNA gene was compared with those in the GenBank (https://blast.ncbi.nlm.nih.gov/Blast.cgi; accessed on 17 December 2021) and Ezbiocloud (http://www.ezbiocloud.net; accessed on 17 December 2021) databases using BLASTN. The similarity values of paired sequences were obtained by the EzBioCloud server. The genomic DNA of strain M9 was sequenced on the Illumina Hiseq sequencing platform (Majorbio, Shanghai, China). The genome assembly was performed using the ABySS 2.1.5 analysis process implemented in SMRT Link (V6.0.0.47841) to get the draft genome [48]. The genome data of strain M9 was uploaded to the Genbank database under the accession number JAEKKD000000000.1.

### 3.5. Bioinformatics Analysis and Structure Prediction of the Alginate Lyases

The putative alginate lyases in strain M9 were predicted by dbCAN meta server (https://bcb.unl.edu/dbCAN2/blast.php; accessed on 17 December 2021) [49]. The signal peptides were predicted using the SignalP5.0 server (http://www.cbs.dtu.dk/services/SignalP/; accessed on 17 December 2021). The theoretical isoelectronic point (pI) and MW were predicted by the ExPASy Server (https://web.expasy.org/compute_pi/; accessed on 17 December 2021) [50]. Domain analysis was performed by blasting at the Conserved Domain Database (https://www.ncbi.nlm.nih.gov/Structure/cdd/wrpsb.cgi; accessed on 17 December 2021) [51]. The sequence identity was determined based on a comparison with the characterized alginate lyases in the CAZy database [52]. The multiple sequence alignment was carried out by ClustalW 2.0.10 (Heidelberg, Germany). The phylogenetic analysis was performed by MEGA 7.0 (Auckland, New Zealand) [53]. The protein structure of AlyM2 was predicted by Alphafold2 (London, UK) [42].

### 3.6. Gene Cloning, Protein Expression and Purification

Genes of alginate lyases without the signal peptides were amplified from the genomic DNA of strain M9 via PCR using the primer pairs with the restriction enzyme sites of NdeI and XhoI (Table 3) and cloned into the expression vector pET-22b between these restriction sites along with a C-terminal His tag. The constructed expression vectors were transferred into *E. coli* BL21 (DE3). The recombinant *E. coli* BL21 (DE3) strains were cultured in LB medium and induced by adding 0.3 mM isopropyl b-D-1-thiogalactopyranoside (IPTG) at 15 °C for 16 h. After cultivation, cells were collected and disrupted by sonication in the buffer containing 50 mM Tris-HCl buffer (pH 8.0) and 100 mM NaCl. The recombinant proteins in the cell extract were purified by nickel–nitrilotriacetic acid resin (Qiagen, Germantown, Germany). The purified proteins were analyzed by sodium dodecyl sulfate polyacrylamide gel electrophoresis (SDS-PAGE).

### 3.7. Biochemical Characterization of AlyM2

The concentration of AlyM2 was determined by the bicinchoninic acid (BCA) protein assay kit (Thermo, Waltham, MA, USA), with bovine serum albumin (BSA) as the standard. The activities of AlyM2 on PM, PG, PMG, and sodium alginate were measured by the ultraviolet absorption spectrometry method [54]. Briefly, a 200 μL mixture containing 60 μg/mL enzyme and 2 mg/mL substrate in 50 mM Tris-HCl (pH 8.0) and 0.5 M NaCl was incubated at 30 °C for 10 min. The reaction was then terminated by boiling for 10 min. The increase in the absorbance at 235 nm (A_235_), resulting from the release of unsaturated uronic in the mixture, was monitored. One unit (U) of enzyme activity was defined as the amount of enzyme required to cause an increase of 0.1 at 235 nm per minute.

The optimum temperature for AlyM2 activity was determined at a range of 20 to 50 °C at pH 8.0. The optimum pH for AlyM2 activity was determined at 30 °C in the Britton-Robinson (B-R) buffer ranging from pH 5.0 to 10.0. B-R buffer was prepared with boric acid, acetic acid, and phosphoric acid, all with final concentration of 0.04 M in an aqueous solution, and was adjusted to different pH with 0.2 M NaOH. The effect of NaCl on AlyM2 activity was determined at NaCl concentrations ranging from 0 to 2.0 M. The substrate specificity of AlyM2 was determined with PM, PG, PMG, and alginate sodium at 30 °C in 50 mM Tris-HCl (pH 8.0) and 0.5 M NaCl.

### 3.8. Degradation Product Analysis

The end degradation products of alginate lyases on sodium alginate were analyzed by using 2 mg/mL substrate and excess enzyme (AlyM1, 132 μg/mL; AlyM2, 80 μg/mL; AlyM2, 144 μg/mL; AlyM5, 58 μg/mL). The degradation reaction was carried out in a buffer containing 50 mM Tris-HCl (pH 8.0) and 0.5 M NaCl at 30 °C for 12 h, after which the kind and amount of AOS in the products were no longer changed even if the reaction time was further extended. The end degradation products of AlyM2 on PM, PG, and PMG were obtained with the same method. The resultant degradation products were analyzed by gel filtration chromatography on a Superdex Peptide 10/300 GL column (GE Healthcare, Pittsburgh, PA, USA) using high performance liquid chromatography (HPLC). The flow rate was 0.3 mL/min with 0.2 M ammonium hydrogen carbonate as the running buffer. Elution was monitored at 210 nm using a UV detector. LabSolutions 6.108 (Shanghai, China) software was used for online monitoring and data analysis. The proportion of trisaccharides in the product of AlyM2 was calculated based on the peak area of the trisaccharides divided by that of all the products in the gel filtration chromatogram. The effect of degradation time on the products of AlyM2 were analyzed by the TLC method. A 200 μL mixture containing 80 μg/mL AlyM2 and 2 mg/mL substrate (PM, PG, PMG, or sodium alginate) in the buffer containing 50 mM Tris-HCl (pH 8.0) and 0.5 M NaCl was incubated at 30 °C for 0, 3, 6, or 12 h. Then, the degradation products and saturated oligosaccharides standards at DP1-DP5 were separated using a solvent system of 1-butanol/acetic acid/water (4:6:1, *v/v*) and visualized by heating the TLC plates at 90 °C for 15 min after spraying with 10% (*v/v*) sulfuric acid in ethanol.

To determine the production of trisaccharides from sodium alginate degradation by AlyM2, a 500 mL reaction mixture containing 1 g sodium alginate and 40 mg AlyM2 in the buffer containing 50 mM Tris-HCl (pH 8.0) and 0.5 M NaCl was incubated at 30 °C for 6 h. The production of trisaccharides was determined by gel filtration on a Superdex Peptide 10/300 GL column at a flow rate of 0.3 mL/min using ultrapure water as the running buffer. Elution was monitored using a refractive index detector (RID). LabSolutions software was used for online monitoring and data analysis. A standard curve of trisaccharide concentration versus peak area was drawn by using different concentrations of commercial saturated mannuronate trisaccharide as the standard. The trisaccharide amount released from sodium alginate by AlyM2 was determined based on the standard curve.

## 4. Conclusions

Although many alginate lyases have been studied, only a few lyases have been shown to have potential in the preparation of AOS with specific DPs. In this study, we identified and characterized an alginate lyase, AlyM2, that has potential in the preparation of different alginate trisaccharides. AlyM2 was identified from an alginate-degrading strain *Pseudoalteromonas arctica* M9 that was screened out from *Sargassum*. AlyM2 is a PL6_3 alginate lyase with low sequence identity to the characterized lyases and does not possess the conserved catalytic amino acids reported in the other PL6 lyases. AlyM2 is an endotype lyase, exhibiting the highest activity at 30 °C, pH 8.0, and 0.5 M NaCl. It mainly produces trisaccharides from PM, PG, PMG, or sodium alginate, and trisaccharides account for approximately 80% of the products. The production of trisaccharides from sodium alginate degradation is 588.4 mg/g. These results demonstrated that AlyM2 is a novel alginate lyase that likely has good potential application in the production of different alginate trisaccharides such as trimannuronate from PM, triguluronate from PG, and a mixture of trisaccharides from PMG and sodium alginate. The structures of these trisaccharides and their potential bioactivities await further investigation.

## Figures and Tables

**Table 1:** Bioinformatics analysis of the alginate lyases of strain M9.

Enzymes	Family	Length (aa)	MW (kDa)	PI	Signal Peptide	Sequence Comparison	
						Sequence Identity ^#^	Reference Alginate Lyase
AlyM1	PL7	365	40.48	7.17	Yes	53.9%	EAP94925.1
AlyM2	PL6	910	94.93	3.92	Yes	28.3%	AFS36385.1
AlyM3	PL17	736	81.94	5.92	Yes	62.6%	AHW45238.1
AlyM4	PL6	752	82.73	8.14	Yes	60.5%	AFS36376.1
AlyM5	PL18	395	42.49	4.89	Yes	93.2%	ACK10595.1

^#^ The sequence identity was obtained by sequence comparison with a characterized alginate lyase whose accession number is listed in the right column.

**Table 2:** Comparison of the properties of the alginate lyases generating trisaccharides.

Enzyme	Source	PL Family	MW (kDa)	Substrate Specificity	Degradation Products	Reference
AlyM2	*Pseudoalteromonas arctica* M9	PL6_3	94.93	PM, PG, PMG and sodium alginate	trimers and tetramers; trimers are of great majority	This study
Alg7A	*Vibrio* sp. W13	PL7	57.66	PM, PG, PMG and sodium alginate	dimers, trimers, and tetramers; mainly trimers	[32]
AkAly28	*Aplysia kurodai*	-	~28	PM	manuronates at DP2-DP5, mainly DP3	[39]
AlyF	*Vibrio splendidus* OU02	PL6_1	59.03	PG	mainly triguluronate	[38,44]

-, no data available.

**Table 3:** Primers used in this study *^a^*.

Primer	Sequence
27-F	5′-AGAGTTTGATCCTGGCTCAG-3′
1492-R	5′-GGTTACCTTGTTACGACTT-3′
AlyM1-F	5′-AAGAAGGAGATATACATATGTGTTCAAGTACCCAAAGCAC-3′
AlyM1-R	5′-TGGTGGTGGTGGTGCTCGAGTTTTGTTGGCGGTGTCGCTG-3′
AlyM2-F	5′-AAGAAGGAGATATACATATGTGTGACACAAACTCAAACAA-3′
AlyM2-R	5′-TGGTGGTGGTGGTGCTCGAGATCGTTTTGTATTTTCCATG-3′
AlyM3-F	5′-AAGAAGGAGATATACATATGGCGCACCCAAACTTAGTAAT-3′
AlyM3-R	5′-TGGTGGTGGTGGTGCTCGAGCTCCTGATTATTGTTCATCA-3′
AlyM4-F	5′-AAGAAGGAGATATACATATGAAAGATTATTTTGTAGAAAG-3′
AlyM4-R	5′-TGGTGGTGGTGGTGCTCGAGCCCTGCCTTATTTAAAATGT-3′
AlyM5-F	5′-AAGAAGGAGATATACATATGGCAACTGTTAATAATGCTGG-3′
AlyM5-R	5′-TGGTGGTGGTGGTGCTCGAGGTTATACGCGTAAAACTAAC-3′

*^a^* Restriction enzyme sites used for cloning are underlined.

## Data Availability

The genome data of strain M9 has been submitted to the NCBI Genbank database under the accession number JAEKKD000000000.1. It can be found here: https://www.ncbi.nlm.nih.gov/nuccore/JAEKKD000000000.1/; accessed on 17 December 2021.
